# Reproductive function and pregnancy outcomes in women treated for idiopathic hyperprolactinemia: A non-randomized controlled study

**DOI:** 10.18502/ijrm.v18i12.8025

**Published:** 2020-12-21

**Authors:** Khatuna Sokhadze, Sophio Kvaliashvili, Jenaro Kristesashvili

**Affiliations:** ^1^Faculty of Medicine, Ivane Javakhishvili Tbilisi State University, Tbilisi, Georgia.; ^2^Medical Clinic “Health House”, Tbilisi, Georgia.; ^3^Center for Reproductive Medicine “Universe”, Tbilisi, Georgia.

**Keywords:** Hyperprolactinemia, Pregnancy outcome, Bromocriptine, Dydrogesterone.

## Abstract

**Background:**

Few studies have focused to determine the peculiarities of the course of pregnancy and pregnancy outcomes after treatment in women with idiopathic hyperprolactinemia.

**Objective:**

To determine the peculiarities of the course of pregnancy and pregnancy outcomes in women treated for idiopathic hyperprolactinemia, with history of infertility and/or recurrent pregnancy loss.

**Materials and Methods:**

A non-randomized controlled study was conducted at the Center for Reproductive Medicine “Universe" and Medical Clinic “Medhealth” during 2016-2018, involving 96 women with idiopathic hyperprolactinemia, aged 20-44 yr with infertility and/or a history of recurrent pregnancy loss. Prolactin (PRL), follicle-stimulating hormone, luteinizing hormone, estradiol (E2), free testosterone, and progesterone were studied in blood serum using immunoassay analysis method. Before the occurrence of pregnancy, hyperprolactinemia was treated with bromocriptine. Dydrogesterone was used to support the luteal phase.

**Results:**

PRL levels decreased significantly and normalized within two-five months, regular menstrual cycle was restored in two-four months, ovulation was restored in three-seven months, and pregnancy was achieved in three-fourteen months. E2 and progesterone levels increased significantly (p < 0.001). Prior to the treatment, significant negative correlation between PRL and E2 (r = -0.386, p = 0.007), PRL and progesterone (r = -0.420, p = 0.003) was detected. Threatened early abortion prevailed among pregnancy complications. Pregnancy loss in the first trimester was recorded in 3.12% of cases.

**Conclusion:**

Pregnancy outcomes in women with idiopathic hyperprolactinemia are improved by prolonged and continuous treatment with bromocriptine before pregnancy and administration of dydrogesterone in support of the luteal insufficiency.

## 1. Introduction

Prolactin (PRL) is anterior pituitary hormone, which significantly influences woman's reproductive function (1). In cases of abnormal increase of blood PRL levels, often an ovulation disorder, luteal phase deficiency, and chronic anovulation are present that lead to fertility disorder (2, 3). Moreover, the prevalence of hyperprolactinemia in women with reproductive disorders varies between 9 and 17% (4).

Besides infertility, recurrent pregnancy loss (RPL) is also a significant problem, which ranges from 2-5% among the couples (5). Endocrine factors are among important causes of RPL, which also include hyperprolactinemia (6), though its role in RPL is not completely specified. One of the mechanisms of negative impact of hyperprolactinemia on reproductive function is considered to be a luteal phase deficiency developed on its background (7). Luteal phase deficiency is considered to be a condition of decreased progesterone secretion, which is essential for both the secretory transformation of endometrium and normal embryo implantation and growth (8). There are three types of hyperprolactinemia: mild hyperprolactinemia, where PRL level varies from 25-50 ng/ml, moderate hyperprolactinemia, where PRL level is between 50 and 100 ng/ml, and higher PRL levels > 100 ng/ml.

Besides, in about 40% of moderate hyperprolactinemia, the cause of PRL hypersecretion cannot be determined. In such cases, it is qualified as idiopathic hyperprolactinemia (9-11). Medication treatment with dopamine agonists is considered as a first-line treatment for idiopathic hyperprolactinemia. Currently, the three most widely used medications in this group are bromocriptine (non-selective agonist), cabergoline (selective agonist), and quinagolide (selective agonist). They restore ovulation and fertility (12). Bromocriptine is preferred drug among women who want to give birth. It improves pregnancy outcomes in women with history of RPL and idiopathic hyperprolactinemia (13). Although bromocriptine is considered as safe at early pregnancy stages, its safety for the whole term of pregnancy is not yet determined (2). Treatment with bromocriptine has good outcomes; however, in 75% of cases the PRL levels increase again after the discontinuation of treatment (14). It is important for infertility to maintain low PRL levels from 10-12 months (2).

To date there are very few studies (2, 6, 12, 13) focused on determining the peculiarities of the course of pregnancy and pregnancy outcomes after treatment in women with hyperprolactinemia. There are no sufficient evidences on the effectiveness of dopamine agonists in relation to improved pregnancy outcomes in women with idiopathic hyperprolactinemia and RPL (12). It is important to conduct research in this direction. Therefore, the objective of our study is to determine the peculiarities of the course of pregnancy and pregnancy outcomes in women treated for idiopathic hyperprolactinemia, with history of infertility and/or RPL.

## 2. Materials and Methods

A non-randomized controlled study was conducted at the Center for Reproductive Medicine “Universe" and Medical Clinic “Medhealth” between September 2016 and October 2018 and included 96 women aged 20-44 yr (average age 29.94 ± 4.59 yr), diagnosed with infertility and/or two or more pregnancy loss before their inclusion in the study, and those who had mild and moderate increase in PRL level (39.0 ng/ml - 68.0 ng/ml; Average 49.46 ± 8.77). The inclusion criteria of the study were women with infertility and/or RPL with idiopathic hyperprolactinemia, who had not received treatment for six months prior to inclusion in the study. However, other etiological factors (such as anatomical, genetic, immunological, infectious, and other endocrinological factors) of infertility and/or RPL were considered as the criteria for non-inclusion in the study. Additionally, those with hyperprolactinemia due to organic damages, polycystic ovary syndrome and congenital adrenal hyperplasia, primary hypothyroidism, or due to pharmacological medications were also excluded.

Prior to inclusion in the study, all women were tested on thyroid-stimulating hormone and free thyroxine levels in blood serum and underwent ultrasound examination of thyroid gland. Based on the results of these studies, hyperprolactinemia developed due to primary hypothyroidism was excluded. PCOS, as one of the most prevailed mild and moderate causes of hyperprolactinemia, was excluded based on the Rotterdam 2003 Consensus Diagnostic Criteria for PCOS. Congenital adrenal hyperplasia was excluded on the basis of hormonal investigation of 17-hydroxyprogesterone and dehydroepiandrosterone sulfate. Organic causes of hyperprolactinemia were excluded with the study of hypothalamic-pituitary area with the use of magnetic resonance imaging with contrast agent.

Women involved in the study were divided into three groups: group (I) 63 women with primary infertility; Group (II) 15 women with RPL; and Group (III) 18 women with secondary infertility. Group III was further divided into subgroups: subgroup (A): 7 women with the history of physiological delivery and subgroup (B): 11 women with the history of one spontaneous abortion. Archival material were considered for control-medical records of 78 women with idiopathic hyperprolactinemia, who were not treated with bromocriptine continuously before pregnancy, stopped taking it immediately as the ovulation was restored, and did not receive dydrogesterone neither before nor during pregnancy. During the study of the history, attention was focused on peculiarities of menstrual and reproductive functions. Among the objective data, we focused on body mass index, g*alactorrhea*, and dermatopathies (acne, seborrhea, and hirsutism). All women underwent gynecological examinations.

Using the immunoassay analysis method (ELISA) (with the use of Rayto Rt-2100 C Microplate Rider), the following hormones in the blood serum were studied: before starting the treatment, the serum PRL levels were determined twice, on the 2nd and 3rd days of menstrual cycle: PRL (initially once a month before the restoration of normal levels and then once every three months), follicle-stimulating hormone (FSH), luteinizing hormone (LH), estradiol (E2), free testosterone (FT) (initially before treatment and after PRL levels were normalized), progesterone level was determined on the 22^nd^ and 23^rd^ days of cycle (initially before treatment and after PRL levels were normalized). Prior to the treatment, in cases of amenorrhea and oligomenorrhea, hormonal study was conducted on the days mentioned earlier, following the menstrual induction. With the use of study device (Voluson E8) ultrasound examination of small pelvic cavity organs was performed. Ovulation was identified with ultrasound examination through follicle monitoring and determination of progesterone on the 22^nd^ and 23^rd^ days of cycle.

Further, women included in the study were treated with bromocriptine continuously until the occurrence of pregnancy. Treatment was started with 1.25 mg daily. Dose was increased gradually by 1.25 mg every week, until PRL reached normal levels (to a median of 5.0-7.5 mg/day in two intakes). In order to maintain long-term normal PRL levels, bromocriptine was prescribed in minimal doses (1.25 mg/day in two intakes) for three menstrual cycles after restoration of ovulation against the background of barrier contraception. In order to support luteal phase, 10 mg dydrogesterone was prescribed for peroral administration for at least three menstrual cycles from 14^th^ through 25^th^ day. After the discontinuation of barrier contraception treatment was continued with both bromocriptine and dydrogesterone in the same doses until the occurrence of pregnancy. For the prevention of luteal deficiency, women were given dydrogesterone 10 mg daily during the first trimester of pregnancy. Women with the history of RPL, dydrogesterone 10 mg was given twice a day until the 20^th^ week of pregnancy with gradual dose decrease. In cases of threatened abortion, they were given a single dose (40 mg) of dydrogesterone, followed by 10 mg every 8 hr until symptoms disappeared. All pregnancy complications and pregnancy outcomes were accurately recorded.

### Ethical consideration

The study was approved by the Ethics Committee of the Center for Reproductive Medicine “Universe”, Tbilisi, Georgia (No3/2016). Written informed consent to participate in the study were obtained from all patients.

### Statistical analysis

While the continuous variables are expressed as Mean ± SD, the categorical variables are expressed as frequencies and percentages. Paired Samples *t* test was used to compare the continuous variables, and a correlation analysis between continuous variables of PRL and other hormones (FSH, LH, FT, E2, and progesterone) was performed using the Pearson's correlation test. All statistical analyses were performed using the Statistical Package for the Social Sciences, version 22.0, SPSS Inc., Chicago, Illinois, USA.

## 3. Results

About 78.12% (n = 75) of the women studied had a history of timely menarche, 21.87% (n = 21), while delayed menarche and premature menarche was not observed in any of the cases; 73.96% (n = 71) had oligomenorrhea, 4.17% (n = 4) oligomenorrhea, and 14.58% (n = 14) secondary amenorrhea. While only 7.29% (n = 7) had regular menstrual cycle, 71.43% (n = 5) had anovulation and 28.57% (n = 2) had luteal phase deficiency. Besides, galactorrhea occurred in 30.21% (n = 29). The body mass index was found within the normal range (18.5-24.0) in 91.67% (n = 88), while 8.33% (n = 8) were overweight (25.1-27.3). Acne was detected in 16.67% (n = 16), of which 81.25% (n = 13) had mild and 18.75% (n = 3) moderate acne. Hirsutism was not detected in any case. The duration of infertility among women involved in the study made on average 2.55 ± 0.78 years (1.5-4.0). Normal PRL level was restored in an average of 3.78 ± 1.58 months (2.0-5.0) after the onset of treatment. Menstrual cycle was regulated in an average of 3.08 ± 0.79 months (2.0-4.0). Ovulation was restored in an average of 5.85 ± 1.60 months (3.0-7.0). Pregnancy was achieved in an average of 6.57 ± 2.15 months (5.0-14.0). Continuous treatment with bromocriptine for achievement of pregnancy took an average of 6.89 ± 2.18 months (3.0-14.0).

Treatment resulted in significant decrease in PRL levels and normalization of its concentration. E2 and progesterone levels increased significantly (p < 0.001). While the FT levels decreased significantly (p < 0.001), it was within the normal range before and after treatment. FSH and LH levels were within the normal range before treatment and did not change significantly after the treatment (Table I).

Correlations between PRL and other hormones were detected. Before treatment, FSH, LH, E2, and progesterone showed reliable negative correlation with PRL (Table II).

Among the studied women, threatened early abortion prevailed among the complications of pregnancy - 11.45% (n = 11). Its highest rate was recorded in women with RPL and reached 20% (n = 3). While among women with secondary infertility and a history of one spontaneous abortion, threatened abortion was observed in 18.2% (n = 2), it was observed in 9.5% (n = 6) of the women with primary infertility. In women with secondary infertility with the history of physiological delivery, no case of threatened early abortion was detected. Threatened preterm labor was recorded in 4.17% (n = 4) of women involved in the study and all these occurred in the group of women with primary infertility (6.3%) on week 25-28 of pregnancy. Placental insufficiency occurred in 2.08% (n = 2) and both cases were recorded in the group of women with primary infertility (3.17%). Preeclampsia occurred in 2.08% (n = 2). Both cases occurred in the group of women with primary infertility and made up for 3.17%. In both cases, pregnancy was carried to full term and ended in vaginal delivery. Pregnancy loss occurred in 3.12% (n = 3). All the three cases were recorded in group I: two in the fifth week and one in the seventh week of pregnancy. In all other cases, pregnancy ended in a live healthy newborn. About 85.42% (n = 82) of women involved in the study had a vaginal delivery. Cesarean section was performed in 11.46% (n = 11). Cesarean delivery mainly prevailed in group I and the reasons for cesarean section were: abnormal fetal position in four cases, narrow pelvic outlet in four cases, in pelvic varicose veins in one case, and myopia in two cases.

Pregnancy loss rate was significantly low in study group than in the control group. All cases of pregnancy loss in both the study and control groups were reported in the first trimester of pregnancy. Timely delivery rate was significantly higher in the study group than in the control group and preterm labor was reported in the control group only (Table III).

**Table 1:** Mean hormone levels before and during treatment in women with idiopathic hyperprolactinemia (n = 96)


**Hormones**	**Before treatment (initial stage)**	**During treatment (after normalizing prolactin level)**	**** ***T*** ** test**	**P-value**
**PRL**	49.46 ± 8.77	12.95 ± 2.66	38.29	< 0.0001
**FSH**	6.35 ± 2.63	6.85 ± 1.37	-1.73	0.0878
**LH**	6.57 ± 3.37	6.51 ± 0.89	0.17	0.8617
**E2**	13.59 ± 6.19	71.23 ± 12.06	-45.69	< 0.0001
**FT**	0.96 ± 0.53	0.70 ± 0.42	4.04	0.0001
**Progesterone**	3.06 ± 1.17	17.36 ± 2.96	-44.96	< 0.0001
Data presented as Mean ± SD; Continuous variables were compared using Student *t* test PRL: Prolactin; FSH: Follicle-stimulating hormone; LH: Luteinizing hormone; E2: Estradiol; FT: Free testosterone				

**Table 2:** Correlations between prolactin and other hormones in women with idiopathic hyperprolactinemia with the history of infertility and/or recurrent pregnancy loss


**Hormones**		**PRL**
**FSH**		
	**r**	-0.525${}^{**}$
	**p**	< 0.001
**LH**		
	**r**	-0.434${}^{**}$
	**p**	0.002
**E2**		
	**r**	-0.386${}^{**}$
	**p**	0.007
**FT**		
	**r**	0.076
	**p**	0.610
**Progesterone**		
	**r**	-0.420${}^{**}$
	**p**	0.003
Correlation analysis between categorical variables was performed by Spearman's correlation analyses, p-value < 0.05 was considered as statistically significant, **p < 0.01 PRL: Prolactin; FSH: Follicle-stimulating hormone; LH: Luteinizing hormone; E2: Estradiol; FT: Free testosterone		

**Table 3:** Evaluation of pregnancy outcomes in study and control groups


**Groups**	**Miscarriage abs. N and %**			**Preterm labor**	**Timely delivery**		
	**I trim**	**II trim**	**All**	**Vaginal delivery**	**Cesarean delivery**	**All**
**Study group**	3 (3.12%)	- 3 (3.12%)	- 82 (85.42%)	11 (11.46%)	93 (96.88%)
**Control group**	14 (17.95%)	- 14 (17.95%)	3 (3.85%)	54 (69.23%)	7 (8.97%)	61 (78.20%)
$\chi^{2}$	10.73	10.73	3.76	6.61	0.29	14.745
**p**	0.002	0.002	0.053	0.011	0.593	<0.001
Data presented as n (%); categorical variables were compared using the Pearson's $\chi^{2}$							

## 4. Discussion

PRL is known to directly affect the ovaries. It inhibits secretion of estrogen and progesterone. Hyperprolactinemia causes inhibition of luteinization and steroidogenesis in the ovaries (15-17). In our study, patients had a moderate increase in PRL at initial stage. The higher the PRL level, the greater the decrease in E2 and progesterone levels in the blood serum, FSH and LH levels remained within the norm. On the background of moderate hyperprolactinemia 73.96% of women included in our study had oligomenorrhea, 4.17% had oligomenorrhea, 14.58% secondary amenorrhea, and 7.29% regular menstrual cycle, of which 71.43% had anovulation and 28.57% luteal phase deficiency. It should be mentioned that at higher PRL levels more profound menstrual disorders were observed. In the study reported by other authors, the rate of amenorrhea was 35% (17), which is probably due to the higher PRL levels compared to our study.

The study of the prevalence of hyperprolactinemia among young women with menstrual disorders showed that hyperprolactinemia was a relatively common cause of secondary amenorrhea and equaled to 13.8% (18); our treatment significantly reduced and normalized PRL levels. As a result, E2 and progesterone levels were significantly elevated to normal, reflected in the regulation of the menstrual cycle and restoring of ovulation. In our study, continuous treatment with dopamine agonist in women with idiopathic hyperprolactinemia led to the PRL level normalization, menstrual cycle regulation, and occurrence of pregnancy in all cases. According to C. Weil, in patients with idiopathic hyperprolactinemia after treatment with bromocriptine, in 80% PRL levels are normalized, and in the absence of other causes of infertility, pregnancy occurs in 60-80% (2). In our study, high effectiveness of treatment is likely to be explained by its duration: after the normalization of PRL levels, women continued to receive bromocriptine at maintenance doses until the occurrence of pregnancy.

Crosignani indicates that treatment of hyperprolactinemia should be long-term and last for at least one year, as restoration of ovulatory cycles takes time and at administration of dopamine agonists pregnancy usually occurs after six months (2). As generally known, abnormal increase of PRL levels in the blood cause menstrual cycle disorders, anovulation, luteal phase deficiency, infertility, RPL, worsening the pregnancy outcomes (6-8, 19). According to our study, the timing of normalization of blood PRL levels in women with idiopathic hyperprolactinemia almost coincides with the timing of menstrual cycle regulation (two-five months) and ovulation is restored later (three-seven months). According to Majumdar and Sharma, in cases of idiopathic hyperprolactinemia, lowering of PRL levels with continuous treatment with bromocriptine begins earlier, regulation of menstruation and restoration of ovulation occur later and almost coincide with each other. In addition, these durations are less (PRL level start to decrease within first week, and menstruation is regulated and ovulation restored within about one-two months) (4), than that observed in our study. According to the results of our study, prolonged continuous treatment with bromocriptine for three months before and after ovulation was restored on the background of barrier contraception, and afterward till the pregnancy, also inclusion of dydrogesterone in the treatment for support of luteal phase after ovulations was restored for at least three menstrual cycles before and in the first trimester of pregnancy, significantly improved pregnancy outcomes. Such complications of pregnancy as threatened preterm labor, placental insufficiency, and preeclampsia were low.

According to Hirahara *et al*, continuous treatment with bromocriptine until the ninth week of pregnancy of women with idiopathic hyperprolactinemia with the history of RPL significantly reduces the number of spontaneous abortions (13). According to some authors, a secondary luteal deficiency may occur during pathological hyperprolactinemia due to pituitary dysfunction (2, 3, 7, 8). Conditions related to inadequate secretion of progesterone corpus luteum may affect pregnancy outcomes, as sufficient amount of progesterone is essential for secretory transformation of endometrium and normal embryo implantation and growth (6, 8). In our study, before treatment, progesterone level was decreased in women with idiopathic hyperprolactinemia, and after normalization of PRL level, there was a significant increase in its level. Pregnancy loss occurred in three cases. In all other cases, pregnancy resulted in a live healthy newborn. According to Carp, treatment with dydrogesterone (generally progesterone) is somewhat empirical, however, results of systematic review has shown that its use reduces the likelihood of spontaneous abortions by 47% compared to standard managed pregnancies, and overall, it reduces the absolute rate of pregnancy losses by 11% (20). An administration of dydrogesterone also lowers the rate of subsequent pregnancy loss in women with RPL (21). According to other authors also, an administration of oral dydrogesterone improves pregnancy outcomes in women with RPL (22-24). However, there is no data in the literature on how dydrogesterone administration before and during pregnancy improves pregnancy outcomes in women with hyperprolactinemia. If we take into account the consideration, that hyperprolactinemia is characterized by secondary luteal insufficiency, it becomes clear how important it is to treat with dydrogesterone in support of the luteal phase in women with hyperprolactinemia.

## 5. Conclusion

Prolonged and continuous treatment with bromocriptine before the pregnancy and with dydrogesterone before and in the first trimester of the pregnancy led to a significantly high rate of timely delivery in the study group compared to the control group. The rate of miscarriage and preterm labor was significantly low in the study group than in the control group. In both the groups miscarriages were reported in the first trimester of the pregnancy. Pregnancy outcomes in women with idiopathic hyperprolactinemia were improved by prolonged and continuous treatment with bromocriptine before the occurrence of pregnancy and use of dydrogesterone to support the luteal phase before pregnancy and during the first trimester of pregnancy.

##  Conflict of Interest

The authors have no conflict of interest in this study.
